# Implications of mechanosensitive ion channels in the pathogenesis of osteoarthritis: a comprehensive review

**DOI:** 10.3389/fcell.2025.1549812

**Published:** 2025-05-01

**Authors:** Yuelong Zhang, Huangming Zhuang, Xunshan Ren, Panghu Zhou

**Affiliations:** Department of Orthopedics, Renmin Hospital of Wuhan University, Wuhan, China

**Keywords:** osteoarthritis, mechanical strain, mechanosensitive ion channels, Piezo1, transient receptor potential, two-pore domain potassium, transmembrane protein 16, Epithelial sodium channel

## Abstract

Osteoarthritis (OA) is the predominant cause of joint pain and limited mobility in older people, and its prevalence is increasing as the population ages. Given the lack of effective therapeutic interventions, the disability rate associated with OA is a staggering 53%, which significantly affects the wellbeing of those affected and represents a significant social and family financial burden. Consequently, OA has emerged as a pressing social and public health concern globally. Various forms of mechanical strain, such as dynamic compression, fluid shear, tissue shear, and hydrostatic pressure, serve as crucial physical stimuli perceived by chondrocytes. Recent studies indicate that aberrant mechanical loading represents a fundamental risk factor for OA. Upon exposure to mechanical loading, chondrocytes translate mechanical cues into chemical signals primarily via mechanosensitive ion channels, resulting in alterations in cartilage metabolism. Numerous studies have demonstrated the significance of mechanosensitive ion channels in the pathogenesis of OA, suggesting that therapeutic interventions targeting these channels on chondrocytes may offer potential benefits.

## Introduction

Osteoarthritis (OA) is the most common degenerative joint disease that impacts over 500 million individuals globally and stands as a primary contributor to motor impairment in the older section of the population (Cronstein and Angle, 2023). The key pathological characteristics of OA include cartilage degradation, synovial inflammation, subchondral bone restructuring, and osteophyte formation, resulting in symptoms such as joint pain, stiffness, motor impairment, and potential disability (Wei et al., 2023; Wylde et al., 2018). For people with advanced or end-stage OA, the need for an artificial joint replacement places a considerable financial burden on society and families (Katz et al., 2021). Currently, there remains a lack of efficacious pharmaceutical interventions to halt or reverse the advancement of OA. Clinical guidelines advocate the use of pharmacologic treatments such as nonsteroidal anti-inflammatory drugs (NSAIDs), hyaluronic acid, and platelet-rich plasma primarily for pain management and enhancing quality of life, with limited impact on the deceleration of OA progression (Khumaidi et al., 2022; Feng et al., 2024). Various risk factors, such as advanced age, obesity, inflammation, genetic predisposition, physically demanding occupations, trauma, and joint force line abnormalities, contribute significantly to the development and exacerbation of OA (Chen et al., 2017). The precise molecular mechanisms underlying disease progression in OA are not fully understood. With aging, alterations in the composition and mechanical characteristics of the cartilage extracellular matrix (ECM) occur, which leads to abnormal transmission of mechanical stimuli to the chondrocytes (Lotz and Loeser, 2012; Loeser et al., 2012). This aberrant signaling accelerates the advancement of OA. Additionally, traumatic events such as cruciate ligament injuries, articular surface fractures, and meniscus injuries can disrupt joint biomechanics, compromising tissue resilience and load transmission capabilities, thereby increasing the risk of OA by a factor of 3–8 (Bradley et al., 2023). Due to the increased mechanical load on weight-bearing joints, obese individuals are at a higher risk of developing OA. Moreover, adipocytokines cause the joints of obese individuals to remain in a state of chronic low-grade inflammation, thereby accelerating joint degeneration (Shumnalieva et al., 2023). Consequently, aberrant mechanical signaling is a key factor in the progression of OA and the phenotypic changes observed in chondrocytes (Hodgkinson et al., 2022).

Mechanical signals, such as hydrostatic pressure, fluid shear stress, extracellular matrix stiffness, tissue elasticity, and extracellular fluid viscosity, impact various biological processes, including cell proliferation, differentiation, adhesion, migration, and ECM generation (Fernandez-Sanchez et al., 2015). Mechanical signals are integral to the regulation of tissue and organ growth and development, as well as processes such as regeneration, repair, tumor proliferation, invasion, and metastasis (Di et al., 2023). Within cartilage tissue, chondrocytes are enveloped by a specialized ECM that functions to shield them by minimizing friction during movement and distributing mechanical stress evenly across the surface (Vincent and Wann, 2019). In normal physiological circumstances, mechanical signals are crucial for sustaining the equilibrium between chondrocyte-mediated ECM synthesis and degradation, thereby preserving articular cartilage homeostasis. Physiological levels of hydrostatic pressure (5 MPa and 10 MPa) were observed to enhance the expression of proteoglycans, ECM-related proteins, and type II collagen in chondrocytes cultured *in vitro* (Ikenoue et al., 2003). Comparable effects were noted in chondrocytes cultured in three-dimensional environments and in cartilage explants (Parkkinen et al., 1992; Valhmu et al., 1998). Adequate mechanical loading not only mitigates the likelihood of disease and symptoms associated with OA but also plays a crucial role in preserving the structural integrity of cartilage tissue (Manninen et al., 2001). Although articular cartilage can maintain internal balance under normal mechanical loads, when the mechanical load it bears is lower or higher than the normal physiological level (5Mpa–15Mpa), the extracellular matrix of articular cartilage may be damaged. Studies have shown that patients with spinal cord injuries experience a 6% decrease in cartilage tissue volume after 6 months of paraplegia, and immobilization of the ankle joint for 7 weeks following a fracture results in a 6.6% decrease in cartilage thickness. These findings suggest that a lack of mechanical signaling stimulation can have negative effects on the physiological function of cartilage (Vincent and Wann, 2019). Furthermore, aside from the physical degradation of cartilage tissue, excessive mechanical loading can also trigger pathological alterations in chondrocytes via aberrant mechanical signals. Research has shown that repeated exposure to four times the normal physiological mechanical load in bovine chondrocytes cultured *in vitro* can lead to mitochondrial dysfunction, ultimately disrupting the balance between synthetic and catabolic processes in the extracellular matrix of chondrocytes, resulting in osteoarthritis (Coleman et al., 2016). The signaling pathway initiated by chondrocytes in response to 1.5%–4.5% shear strain amplitude at 0.1 Hz for 20 min mirrors that activated by pro-inflammatory cytokines like interleukin-1β and tumor necrosis factor-α (Fitzgerald et al., 2008). High-frequency cyclic stretching deformation (12%–18%) triggers the activation of the chondrocyte nuclear factor kappa-B (NF-κB) signaling pathway, leading to the secretion of pro-inflammatory cytokines and degradation of the ECM (Agarwal et al., 2004; Pulai et al., 2005). Large peak stress (12–23 Mpa) also induces chondrocytes to produce vascular endothelial growth factor, which in turn stimulates the secretion of matrix metallopeptidase (MMP) 1, MMP3, MMP13, and a disintegrin and metalloproteinase with thrombospondin motifs (ADAMTS5), resulting in cartilage matrix degradation (Kurz et al., 2005).

**FIGURE 1:**
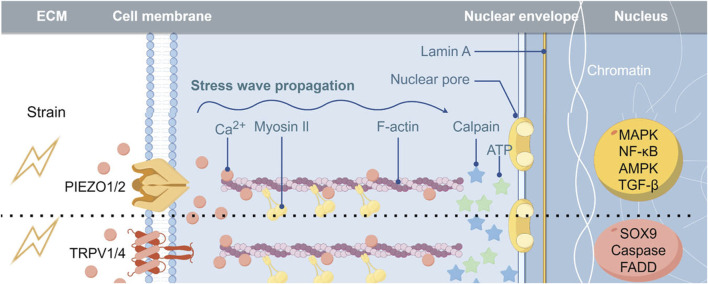
Implications of Mechanosensitive Ion Channels in the Pathogenesis of Osteoarthritis Abbreviations: MAPK, mitogen-activated protein kinase; FADD, Fas-associating protein with a novel death domain.

Chondrocytes exhibit the ability to respond to and transmit mechanical signals through various mechanisms. Specifically, the structural alterations of the cartilage extracellular matrix in response to mechanical stimuli result in the release of growth factors, including fibroblast growth factor, bone morphogenetic proteins, and transforming growth factor β (TGF-β), which subsequently activate intracellular signaling pathways by binding to receptors on the chondrocyte membrane (Vincent, 2013; Martin et al., 2002; Guilak et al., 2006). Additionally, mechanical signals can induce integrin activation via interactions with the extracellular matrix, facilitating the transmission of external mechanical cues into the cell (Gracey et al., 2020). Recent studies have shown that in degenerative osteoarthritis, the stiffness of the pericellular matrix (PCM) is reduced. Chondrocytes can sense and distinguish different PCM stiffness levels through various mechanosensitive ion channels (Du et al., 2022). Utilizing 3D models that can accurately simulate changes in PCM stiffness *in vitro* can more precisely demonstrate this point (Du et al., 2024). Primary cilia, along with cellular signaling components like the mechanical load sensing receptor Gremlin1, have been demonstrated to play a role in transducing mechanical signals (Chang et al., 2019; Fang et al., 2020). Furthermore, mechanosensitive ion channels are membrane proteins that enable cells to detect and react to mechanical signaling stimuli, converting them into electrical signals through ion transport, thereby activating downstream signaling pathways. The current consensus suggests that the mechanical signaling mechanism facilitated by mechanosensitive ion channels represents the most rapid conduction system identified in living organisms thus far, and is integral to the response of chondrocytes to external mechanical stimuli (Xu et al., 2020). Consequently, this review focuses on elucidating the functions and mechanisms of diverse mechanosensitive ion channels in chondrocyte metabolism and OA and investigates the potential effectiveness and importance of targeting mechanosensitive ion channels for OA intervention (Figure 1).

### Piezo type mechanosensitive ion channel

The Piezo family, primarily composed of Piezo1 and Piezo2, was initially discovered in 2010 (Coste et al., 2010). This family is prominently found in tissues that are frequently subjected to mechanical stimuli, such as the colon, kidney, skin, bladder, lung, and cartilage (Lai et al., 2022; Sun et al., 2024; Ran et al., 2023; Choi et al., 2022; Lam et al., 2023). The Piezo family exhibits responsiveness to a variety of mechanical stimuli, including compressive loading, tensile loading, ultrasonic loading, shear stress, matrix roughness, and matrix stiffness (Wu et al., 2017; Jiang et al., 2022; Yang et al., 2022; Hilscher et al., 2019). Piezo1 and Piezo2 are comprised of approximately 2,500–2,700 amino acids arranged in a homotrimer configuration, featuring a structural resemblance to three propeller blades connected to a central ion-conducting pore (Saotome et al., 2018). This blade-like structure is integrated into the cell membrane, enabling the detection of local tension within the membrane and facilitating mechanical signaling through leverage (Zhao et al., 2018). Activation of the Piezo family enables the non-selective transport of Ca^2+^, Mg^2+^, Mn^2+^, Ba^2+^, K^+^, and Na^+^ cations, with a particular affinity for Ca^2+ 40^. When exposed to mechanical signals, the Piezo family promotes the influx of Ca^2+^ into cells and triggers the activation of Ca^2+^-sensitive signaling molecules, such as calpain, as well as the release of ATP, thereby regulating cellular physiological processes (Wei et al., 2019; Li et al., 2015).

Piezo family-mediated mechanosignaling plays a crucial role in organismal survival, as evidenced by the lethal consequences of overall knockout of either Piezo1 or Piezo2 in mice. This lethality may be attributed to impaired development of the circulatory system resulting from Piezo family defects (Li et al., 2014; Ranade et al., 2014). In the respiratory system, Piezo1 facilitates the breakdown of vascular endothelial cadherin through activation of calpain, thereby contributing to the maintenance of tight junctions in lung endothelial cells and stabilization of the lung endothelial barrier (Friedrich et al., 2019). Additionally, in the hematological system, Piezo1 is involved in the regulation of erythrocyte permeability and the maintenance of erythrocyte volume homeostasis (Cahalan et al., 2015). Piezo2 is integral to pressure receptors, contributing to various physiological functions including the regulation of blood pressure, vasoconstriction, heart rate control, skeletal development, and sensory conduction (Min et al., 2019; Assaraf et al., 2020; Sánchez-Carranza et al., 2024; Nagel and Chesler, 2022; Villarino et al., 2023; Nie et al., 2024). Mutations in the Piezo family have been linked to several diseases, such as colorectal adenomatous polyposis, dehydrated hereditary stomatocytosis, hemolytic anemia, and Gordon’s syndrome (Andolfo et al., 2013; Fotiou et al., 2015; Spier et al., 2016). Research has shown that abnormal mechanical signals, such as stretching and ultrasound, can induce apoptosis and necrosis by modulating Piezo1 activity (Kim and Hyun, 2023; Wang et al., 2022). Additionally, high-hardness culture substrates have been found to induce myeloid cell senescence through Piezo1 activation of the NF-κB signaling pathway (Wu J. et al., 2022).

Both Piezo1 and Piezo2 were found to be significantly expressed on the surface of chondrocytes, and knockout mice lacking these ion channels exhibited notable defects in articular cartilage. Mice with Piezo1/2 deficiency typically exhibit a lack of cartilage growth and reduced osteoblast differentiation (Zhou et al., 2020). This suggests that Piezo1 and Piezo2 play a crucial role in the development of articular cartilage and the formation of cartilage ECM. Current research on OA has primarily focused on the function of Piezo1, with studies showing that cyclic mechanical stretching can upregulate Piezo1 expression in chondrocytes (Yang et al., 2018). Additionally, higher levels of Piezo1 have been observed in load-concentrated areas of articular cartilage in OA patients (Wang et al., 2022). In addition to aberrant mechanical stimuli, inflammatory signals in OA appear to influence the expression of Piezo1. Elevated Piezo1 expression was observed in an *in vitro* chondrocyte model of OA induced by interleukin-1β, indicating that inflammatory signals may sensitize articular cartilage to mechanical stimuli, rendering it more vulnerable to damage (Lee et al., 2021). Nevertheless, conflicting results exist, as some studies have reported no change in Piezo1 expression in OA rats (Ikeda et al., 2021). Furthermore, the activation of Piezo1-mediated pathological signaling is a current focal point in OA research. The knee joint local activation of Piezo1 via intra-articular injection of Yoda1 exacerbates OA lesions induced by DMM in adult mice (Gan et al., 2023). Piezo1 senses mechanical stress through its Ca^2+^ channel activity and promotes chondrocyte senescence, wherein MAPK and NF-κB activation are two critical pathways responsible for responding to Piezo1 activation and facilitating the production of IL-6 and IL-1β, respectively (Liu et al., 2023). Additionally, Piezo1 has been found to contribute to articular cartilage degradation through TGF-β in non-weight-bearing joints (Wu C. B. et al., 2022). In conclusion, Piezo1 is a crucial ion channel involved in the growth and maintenance of articular cartilage and extracellular matrix stability, as well as in the progression of OA through aberrant mechanical loading-induced signaling pathways.

Current research on drug interventions targeting Piezo1 has primarily centered on Gsmtx4, a Piezo1-specific inhibitor derived from spider venom that effectively suppresses Piezo1 activity (Matute et al., 2020). Multiple studies have shown the therapeutic benefits of intra-articular administration of Gsmtx4 in treating OA, the mechanisms encompass the inhibition of Piezo1-mediated cartilage apoptosis, the Piezo1 channel facilitates calcium influx, inducing GPX4-regulated chondrocyte ferroptosis in osteoarthritis, and other pathological manifestations (Wang et al., 2022; Ren et al., 2023). Furthermore, Gsmtx4 has demonstrated efficacy in not only decelerating the progression of OA but also alleviating mechanically-induced pain mediated by Piezo1 activation in OA (He et al., 2017). Recently, there has been a gradual development of other drugs with Piezo1 inhibitory properties. Research has indicated that the endogenous prosurvival factor urocortin-1 can selectively block Piezo1 activity, thereby inhibiting intracellular calcium accumulation and providing protective effects against acute cartilage injury (Jones et al., 2022; Lawrence et al., 2017). Moreover, it was demonstrated that the antimalarial medication artemisinin can mitigate cartilage damage resulting from medial meniscus instability in mice (Gan et al., 2023) Despite the beneficial therapeutic outcomes observed in numerous *ex vivo* and *in vivo* investigations, there remains a paucity of clinical data supporting the use of Piezo1-targeted intervention regimens for treating OA patients with associated medications.

### Transient receptor potential (TRP) ion channel

The TRP ion channel family, identified in 1969, comprises a group of multimodal ion channels capable of detecting chemical toxicity and physico-mechanical stimuli (Cosens and Manning, 1969; Chen et al., 2020). The TRP ion channel family found on the surface of mammalian cells can be classified into distinct subclasses, including TRPC, TRPV, TRPM, TRPN, TRPA, TRPP, and TRPML, based on variations in amino acid sequence and topology (Zhang et al., 2023). These channels typically exist as homotetramers and possess six transmembrane structural domains, with the sixth domain serving as a gate structure that regulates the inward flow of Ca^2+^-dominated cations (Zhang et al., 2023). The TRP ion channel family demonstrates synergistic interaction with the Piezo ion channel family through a shared second messenger (G protein released upon their activation) (Zhao and MacKinnon, 2023). However, the TRP ion channel family appears to exhibit a greater emphasis on responding to physiological mechanosignals (Du et al., 2020; Servin-Vences et al., 2018). This family of ion channels exerts influence on various signaling pathways, notably the MAPK, TGF-β, NF-κB, and AMP-dependent protein kinase pathways, which play critical roles in a multitude of physiological and pathophysiological processes, including cell proliferation, metabolism, differentiation, and apoptosis (Zhang et al., 2023). Research has identified the presence of 19 TRP ion channels within the subclasses of TRPV, TRPC, and TRPM in articular cartilage (Halonen et al., 2023). Current investigations in the field of OA have primarily focused on TRPV4 and TRPV1 within the TRPV subclass (Xu et al., 2020).

The TRPV subclass, characterized by a 40% sequence similarity and originally described in 2006, is currently the most extensively studied member of the TRP family in bone and cartilage tissues (Jin et al., 2006). TRPV members play a role in various physiological processes such as intestinal calcium transport, renal calcium reabsorption, osteoblast formation and differentiation, and the regulation of skeletal homeostasis in organisms (Lieben and Carmeliet, 2012). In a physiological context, TRPV4 plays a crucial role in the chondrocyte response to low osmolarity by stimulating the expression of SOX9, a pivotal regulator of chondrogenesis, via the activation of calmodulin phosphatase (Muramatsu et al., 2007; Phan et al., 2009). The absence of TRPV4 in knockout mice resulted in the absence of osmotic alteration-induced Ca2+ signaling in their articular chondrocytes, leading to a gradual decline in bone mineral density and the development of osteoarthritic symptoms, including cartilage degradation and decreased proteoglycan levels (Clark et al., 2010). Research has demonstrated that mutations in the TRPV4 gene can impact human skeletal development to varying degrees, ultimately contributing to the development of inherited arthropathy of hands and feet (Loukin et al., 2011; Lamandé et al., 2011). Mechanical stress applied to chondrocytes also increases TRPV4-mediated Ca^2+^ influx, which induces apoptosis by upregulating caspase-8-dependent apoptotic signaling pathways in chondrocytes (Xu et al., 2019). Furthermore, TRPV4 activation disrupts the interaction between CD44 and hyaluronic acid, leading to dysregulated homeostasis of the articular cartilage matrix (Kobayakawa et al., 2016). The deletion of TRPV4 specifically in cartilage in mice results in a marked reduction in the severity of OA associated with aging (O'Conor et al., 2016). In a similar manner to TRPV4, the function of TRPV1 is essential for cell proliferation, but excessive activation leads to cytotoxic effects, including apoptosis (Somogyi et al., 2015). Additionally, the suppression of TRPV1 alleviates pain symptoms and diminishes joint tissue damage in OA mice by inhibiting extracellular regulation of protein kinase signaling pathway activity (Chen et al., 2009). Furthermore, TRPV1 expression has been identified in synoviocytes and peripheral nerve cells, in addition to chondrocytes. These cells play a crucial role in inflammatory cytokine release and oxidative stress responses, which are closely linked to the progression and severity of pain symptoms in OA (Hu et al., 2008; Wu et al., 2015). Collectively, the function of TRPV subclass ion channels is crucial in the modulation of cartilage tissue development and the progression of OA disease.

Various treatments for OA targeting TRPV subclass ion channels have been developed. Inhibition of TRPV4 activity through the TRPV4-specific inhibitor HC-067047 has been demonstrated to suppress inflammation in the temporomandibular joint in mice (Chen et al., 2013). Additionally, for TRPV4 mutation-induced OA chondrodysplasia, the TRPV4 activators GSK1016790A or 4α-PDD have shown therapeutic efficacy (Clark et al., 2010). Studies on isolated cells have shown that capsaicin and other natural substances can activate TRPV1 (Caterina et al., 2000). The capsaicin-evoked action potential is due to a physical interaction between TRPV1 and anoctamin 1, a calcium-activated chloride channel, which results from the influx of Ca^2+^ through the TRPV1 pore (Takayama et al., 2015). This interaction is relevant to the enhancement of nociception. Nociceptors are polymodal receptors as they respond to stimuli of a heterogeneous nature: mechanical (e.g., high pressure), thermal (too high or too low temperatures), and chemical. The pharmacological activation of TRPV1 by capsaicin significantly impedes macrophage inflammation and subsequent synovitis (Lv et al., 2021). Moreover, TRPV1 is expressed in chondrocytes, and its activation notably protects chondrocytes from ferroptosis and mitigates articular cartilage degradation by upregulating the classical ferroptosis suppressor glutathione peroxidase 4 (GPX4) (Lv et al., 2022). These effects of TRPV1 synergistically attenuate OA progression, offering a promising therapeutic target for OA. However, some studies have shown that activation of TRPV1 channels inhibits mechanosensitive Piezo channel activity by depleting membrane phosphoinositides. These data also suggest that inhibition of Piezo2 channels may contribute to the analgesic effects of capsaicin (Borbiro et al., 2015). Research has demonstrated that intra-articular administration of the TRPV1 antagonist capsaicin effectively reduces pain response in rats with temporomandibular arthritis (Wu et al., 2015). The TRPV1 antagonists SB366791, AMG9810, and ABT-116 have been found to significantly reduce joint pain and inflammatory response in gouty arthritic mice. Capsaicin induces dysfunction of TRPV1-expressing nerve afferent terminals and has been utilized as an analgesic medication in patients with OA (Hoffmeister et al., 2011; Hoffmeister et al., 2014; Cathcart et al., 2012). Inhibition of TRPV4 activity through the TRPV4-specific inhibitor HC-067047 has been demonstrated to suppress inflammation in the temporomandibular joint in mice (Chen et al., 2013). Additionally, for TRPV4 mutation-induced OA chondrodysplasia, the TRPV4 activators GSK1016790A or 4α-PDD have shown therapeutic efficacy (Clark et al., 2010). Therefore, while TRPV subclass ion channels are considered important therapeutic targets for OA, their functional roles in various types of OA must be thoroughly examined to develop personalized treatment plans.

## Discussions

Current research has demonstrated that the expression of Piezo channels has been identified in chondrocytes and cartilage tissues, and it is known that articular chondrocytes detect and respond to mechanical stress through the activation of Piezo channels. Piezo1 is stably expressed in human degenerated chondrocytes and exhibits a time-dependent relationship with mechanical stress. Since chondrocytes in OA patients undergo apoptosis at later stages, there is a pathological connection between the two. It has been confirmed that Piezo1 mediates the response of chondrocytes to mechanical compressive stress, leading to intracellular Ca^2+^ accumulation, which is associated with chondrocyte apoptosis (Delco and Bonassar, 2021). When the Piezo1 ion channel protein is activated by supraphysiological levels of mechanical load, the membrane current rises instantaneously, causing chondrocyte depolarization and activation of voltage-gated calcium channels (VGCC), thereby increasing intracellular Ca^2+^ levels (Lee et al., 2021). This results in mitochondrial dysfunction and ultimately chondrocyte apoptosis. However, TRPV4 is not involved in the amplification of intracellular calcium signaling induced by VGCC activation (Nims et al., 2021). The influx of Ca^2+^ into the cell causes depolarization and regulates kinase cascades, and the activation of apoptosis-related kinase cascades can lead to cell death. Studies have found that the selective Piezo1 agonist Yoda1 increases intracellular Ca^2+^ levels in osteoarthritic chondrocytes and upregulates the expression of matrix metalloproteinases (MMPs), tissue inhibitor of metalloproteinase 2 (TIMP2), bone morphogenetic protein 2 (BMP2), type I collagen α1 (COL1A1), and interleukins (Steinecker-Frohnwieser et al., 2023). Additionally, Yoda1 inhibits the activation of TRPV4 by GSK1016790A, suggesting a potential functional interaction between Piezo1 and TRPV4 in chondrocytes, possibly through direct or indirect physical interactions (Steinecker-Frohnwieser et al., 2023).

Evidence indicates a direct relationship between IL-1 in chondrocytes and increased inflammatory signaling in OA. IL-1α significantly increases Piezo1 expression in human OA chondrocytes, leading to elevated intracellular Ca^2+^ levels (Guilak et al., 2018). The abnormal matrix mechanical microenvironment induced by IL-1β contributes to inflammation and degenerative changes in OA. Overexpression of Piezo1 in chondrocytes results in excessively high intracellular Ca^2+^ concentrations, causing deformation of the actin cytoskeleton and thinning of actin filaments. This amplifies mechanically induced microdamage, thereby increasing the mechanical sensitivity of chondrocytes. Thus, the role of Piezos in mediating mechanotransduction during OA progression is evident. IL-1α enhances Piezo1 expression in chondrocytes, and this enhancement depends on P38 protein kinase as well as transcription factors HVF4 and AFF2/CREBP1. The direct binding of transcription factors to the proximal Piezo1 gene can lead to the expression of inflammatory genes. However, IL-1α does not affect the expression or function of TRPV4 (Lee et al., 2021). Therefore, the mechanotransduction mechanisms in chondrocytes under the synergistic effects of inflammatory signaling and mechanical stimulation may be more complex.

## Conclusion

Ultimately, mechanosensitive ion channels play a crucial role in both the formation of cartilage tissues and the maintenance of articular cartilage equilibrium. The modulation of chondrocyte matrix anabolic activity by physiological mechanical loading via mechanosensitive ion channels is a significant factor in the maintenance of cartilage health. Conversely, the dysregulation of mechanosensitive ion channels can lead to the transmission of abnormal mechanical signals, resulting in altered intracellular ion concentrations and subsequent cartilage damage, ultimately contributing to the progression of OA. Recent research has elucidated the involvement of Piezo and TRP ion channel families in OA pathogenesis, leading to the development of targeted therapeutic interventions aimed at slowing the progression of this degenerative joint disease. Nevertheless, there remain deficiencies in current research. Primarily, clinical investigations of targeted medications are limited to topical administration and pain symptom antagonism, with minimal exploration into the disease progression of OA. Moreover, the current research studies exhibit a limited scope by neglecting the potential mechanosensitivity of diverse ion channels such as the OSCA/TMEM63 and TMC families, and their impacts on chondrocyte expression and function (Wang and Parpura, 2018; Tang et al., 2020). Therefore, it is imperative to further investigate the composition and function of mechanosensitive ion channels in chondrocytes, with particular emphasis on elucidating the molecular mechanisms underlying the mechanobiological transduction mediated by TRPV4 and Piezo. Such research will deepen our understanding of chondrocyte physiology and pathophysiology, and contribute to the identification of novel therapeutic targets for OA.

## Abbreviations

OA, Osteoarthritis; NSAIDs, Nonsteroidal anti-inflammatory drugs; ECM, Extracellular matrix; NF-κB, Nuclear factor kappa-B; MMP, Matrix metallopeptidase; ADAMTS5, A disintegrin and metalloproteinase with thrombospondin motifs; TGF-β, Transforming growth factor β; TRP, Transient receptor potential; K2P, Two-pore domain potassium; TMEM16, Transmembrane protein 16; ENaC, Epithelial sodium channel.
